# Spatial transcriptomics: new dimension of understanding biological complexity

**DOI:** 10.52601/bpr.2021.210037

**Published:** 2022-06-30

**Authors:** Zhuxia Li, Guangdun Peng

**Affiliations:** 1 Ceter for Cell Lineage and Development, CAS Key Laboratory of Regenerative Biology, Guangdong Provincial Key Laboratory of Stem Cell and Regenerative Medicine, Guangzhou Institutes of Biomedicine and Health, Chinese Academy of Sciences, Guangzhou 510530, China; 2 Center for Cell Lineage and Atlas, Bioland Laboratory (Guangzhou Regenerative Medicine and Health Guangdong Laboratory), Guangzhou 510005, China; 3 Institute for Stem Cell and Regeneration, Chinese Academy of Sciences, Beijing 100101, China; 4 University of Chinese Academy of Sciences, Beijing 100049, China

**Keywords:** Spatial transcriptomics, Spatial data analysis, Spatial multi-omics, Histology, Single-cell sequencing

## Abstract

Cells and tissues are exquisitely organized in a complex but ordered manner to form organs and bodies so that individuals can function properly. The spatial organization and tissue architecture represent a keynote property underneath all living organisms. Molecular architecture and cellular composition within intact tissues play a vital role in a variety of biological processes, such as forming the complicated tissue functionality, precise regulation of cell transition in all living activities, consolidation of central nervous system, cellular responses to immunological and pathological cues. To explore these biological events at a large scale and fine resolution, a genome-wide understanding of spatial cellular changes is essential. However, previous bulk RNA sequencing and single-cell RNA sequencing technologies could not obtain the important spatial information of tissues and cells, despite their ability to detect high content transcriptional changes. These limitations have prompted the development of numerous spatially resolved technologies which provide a new dimension to interrogate the regional gene expression, cellular microenvironment, anatomical heterogeneity and cell-cell interactions. Since the advent of spatial transcriptomics, related works that use these technologies have increased rapidly, and new methods with higher throughput and resolution have grown quickly, all of which hold great promise to accelerate new discoveries in understanding the biological complexity. In this review, we briefly discussed the historical evolution of spatially resolved transcriptome. We broadly surveyed the representative methods. Furthermore, we summarized the general computational analysis pipeline for the spatial gene expression data. Finally, we proposed perspectives for technological development of spatial multi-omics.

## INTRODUCTION

Multicellular organisms rely on a variety of cellular events such as cell movement, communication between interacted cells and position-dependent gene expression at distinct microenvironments. Studying these cellular events on the anatomical structure is essential to the understanding of many important biological and pathological questions. In essence, the orchestrated composition of cells and tissues at predefined locations is a prerequisite for the complex functionality of biological system. For example, the steady-state lymph nodes have intrinsic location patterns including cortex, paracortex and medulla, composed by distinct cell types (Stoltzfus *et al*. 2020). The correct position and interaction between immune cell subpopulations are critical for lymph nodes to function well and maintain immune homeostasis. The approaches to systematically survey a cell’s transcriptome in its tissue architecture at genome-wide level become a biological imperative.

Recently, plenty of spatial gene profiling technologies have been developed, aiming to reveal the molecular changes on 2D and 3D tissue samples at given positions and even at single cell or subcellular resolution (Chen *et al*. 2021; Cho *et al*. 2021). The modern age technologies are originated from historical tools that can quantify gene expression and preserve spatial information. Some essential spatial gene atlas and databases during numerous dynamic processes have been accomplished by these historical methods. For example, histological staining using chemical dye such as hematoxylin and eosin, has been performed on tissue samples containing cells of distinct types and positions, and the tissue organization and anatomical structure are visualized under electron or light microscopes (Alturkistani* et al.* 2015). Furthermore, an improvement on histological staining has been achieved by targeting more specific tissue regions or cell subgroups with the application of molecular techniques, for example, genes or gene promoters are fused to fluorescent signals to allow for gene trapping or promotor trapping, which are popular tools to investigate the influence of specific genes during embryonic development (Skarnes *et al*. 1995). Nevertheless, gene trapping and promotor trapping are both random insertions, which means the vector is probably biasedly inserted into some large gene or promoter sequences. Moreover, only one or a few selected genes could be studied at one time. Of note, *in situ* hybridization (ISH) or immunohistochemistry (IHC) is an accurate and effective method, which detects multiple spatial patterns of gene or protein with the aid of nucleic acid probes or antibodies. With the development of sequencing technology in the late 20th century, genomic information of model species was completed, which enhanced the design and synthesis of *in situ* reporters. For instance, fused cosmids with reporter and upstream regulators based on whole genome sequence were constructed to reveal the expression of many development-dependent genes in nematode *Caenorhabditis elegans* (Lynch *et al*. 1995).

While traditional spatial techniques have common unfulfillment-samples and genes that can be simultaneously examined are limited. In addition, these methods with a few predefined genes and a small sampling size may result in the biased descriptions. It is also unclear in which position the whole genome is transcribed on the natural tissue structure, which is indispensable for exploring molecular distributions, discovering novel spatial expression patterns and querying the function of candidate genes in development, diseases and cancer. Therefore, expanding our knowledge on the expression of transcripts meanwhile preserving the morphology of tissues will facilitate the understanding of cell type heterogeneity, cell-cell interaction, cell fate dynamics in normal and abnormal biological contexts. Hence, unbiased and systematic gene profiling of tissues with spatial coordinates requires the implement of spatial transcriptome technologies.

Even though basic principles and capacities of spatial transcriptomic technologies vary widely, methods of transcriptome-level profiling can be roughly classified into three groups according to their space-procuring protocols: technologies based on FISH, technologies based on isolation through microdissection or photoreaction, and array-based technologies.

## TECHNOLOGIES BASED ON FISH

*In situ* hybridization (ISH) was first reported in 1969 by Pardue and Gall to detect the hybrid of DNA and RNA. The main principle of ISH for visualizing target molecules is the detection of hybridization signals of labeled molecule probes including isotope, fluorescence and enzyme by autoradiography or microscopy (Francoz *et al*. 2016). FISH has been the standard approach and the routine procedure in various experimental contexts (Shaffer *et al*. 2017). Powered by ISH, a number of imaging-based RNA sequencing techniques have been developed to generate high-resolution spatial molecular atlas of different organ at different developmental systems at single-cell and subcellular levels (Lohoff 2021).

Nevertheless, there are still drawbacks of these technologies, such as the challenge of complicated image processing and the difficulties to keep the balance between detection efficiency, sensitivity and the detected number of transcripts. Importantly, a large amount of customized costly probes must be designed and synthesized with *a priori* knowledge, which may limit the application and obstruct new findings.

### smFISH

**Figure 1:**
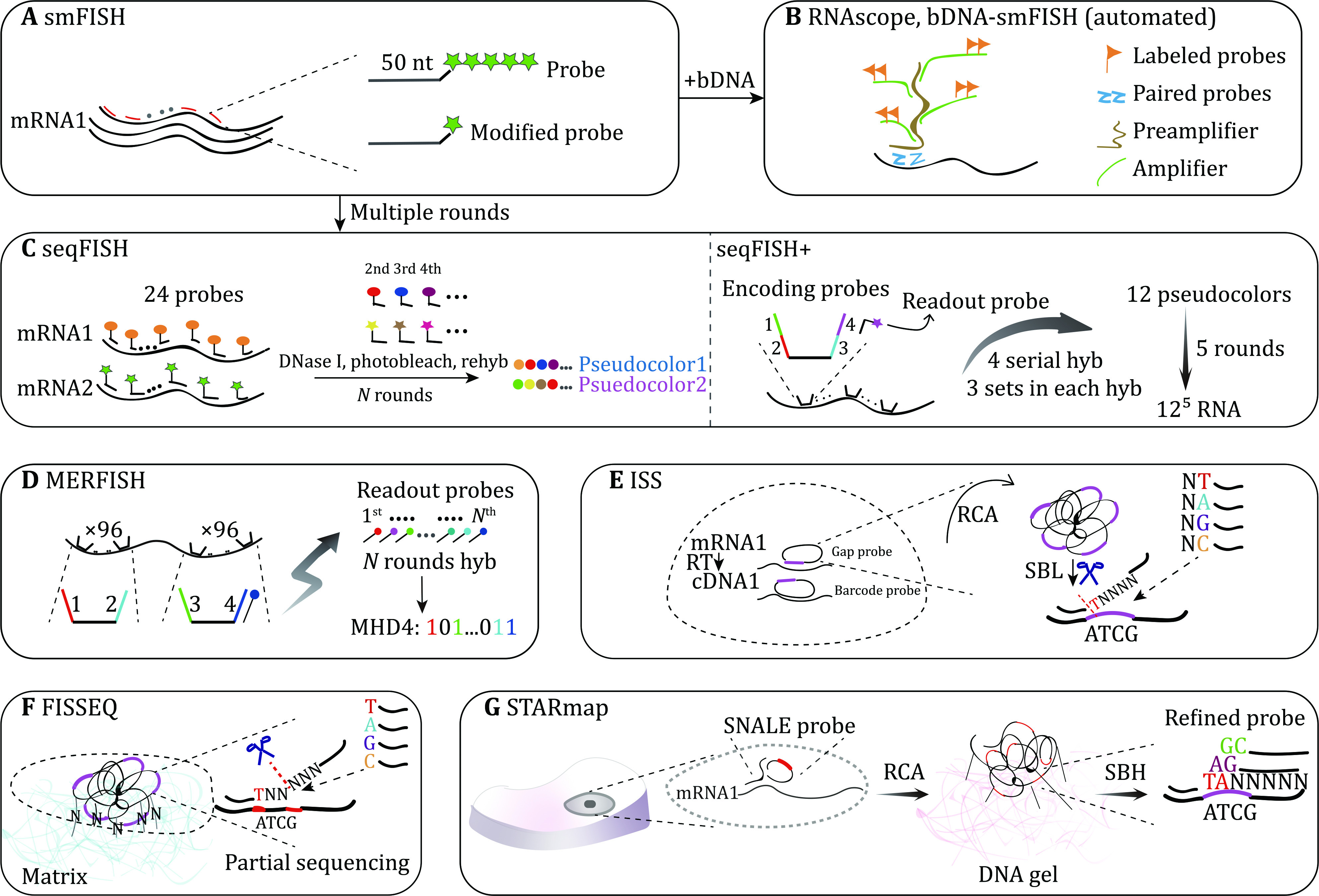
Technologies based on FISH. **A** smFISH: 50 nt probes labeled with the same five fluorescence are bound to targeted mRNA. Improved probes are merely labeled with one fluorescence. **B** RNAscope and bDNA-smFISH: based on smFISH, branched DNA technology is merged to amplify mRNA signals in the background of noise. Probes pair firstly hybridize to specific sequences on mRNA, which provide a site that preamplifier binds. Several amplifiers with multiple labeled probes are bound with preamplifiers. bDNA-smFISH can be completed by automated liquid handling. **C** seqFISH: a series of 24 probes with respectively distinct fluorophores are loaded into individual target mRNA. In the first round, 24 probes with specific color are introduced, then probes are removed by DNase I and photobleached. Identical probes labeled with different fluorophore are rehybridized at the next successive rounds. Finally, each mRNA can be detected as unique composite colors of its probes. seqFISH+ applies encoding probes instead of common probes, which are composed of target complementary sequence and four readout sequences that can bind to distinct colored readout probes. In each four hybridizing rounds, 12 sets of readout probes with just three colors are used to generate 12 pseudocolors representing targeted mRNA. Therefore, after five pseudocolor rounds, each mRNA is marked by a composite color of five pseudocolors. **D** MERFISH: two sets of 96 encoding probes with two readout parts are linked to a particular target. Different fluorescence conjugated readout probes are added respectively in N rounds, decoding mRNA by MHD4 which “1” means hybridizing signal and “0” means no signal. **E** ISS: cDNA reversely transcribed from mRNA is identified by padlock probes. Here two strategies can be used: (1) Probe with a gap at the complementary site is circularized to form target sequence; (2) Probe perfectly binding to mRNA has a barcode on the remaining site. After circularization, multiple target sequences or barcode sequences can be recognized through SBL. **F** FISSEQ: cDNA nanoball can be fixed on the extracellular matrix by ligation of amine-reaction and partially sequenced. **G** STARmap: mRNA of cells residing in thick tissue directly links to SNAIL probes. Primer pairing to target mRNA and padlock probe initiates RCA. Barcodes of nanoball conjugated with DNA gel are then encoded by SBH in which the base pair of sequencing probes are labeled with correspondent color for error correction (SEDAL). MHD4: modified Hamming distance; SBL: sequencing by ligation; SBH: sequencing by hybridization

Compared to the long probes targeting one transcript in ISH, single molecule FISH (smFISH) utilizes a series of shorter fluorescent probes (often 40–50 nucleotides) hybridizing to different sequences of one nucleic acid molecule (Femino* et al*. 1998). Individual small probes are coupled with five fluorophores (such as CY3 and FITC). In this way, the targeted DNA or RNA can be marked with numerous fluorescence, and then imaged by fluorescent microscopy to obtain the information of its high intensity light spots (Fig. 1A). The image information of each gene or mRNA molecule is constrained by deconvolution algorithms into voxels. Other interference voxels introduced by autofluorescent signal and background noise are filtered by setting a threshold. Therefore, smFISH provides the advantages of high-resolution and sensitivity in visualizing and detecting single target molecules. For example, smFISH was described to successfully identify the dynamic snapshots of the transcription initiation, termination, processing and transporting of β-actin mRNA in normal rat kidney cells using oligonucleotide probes targeting to 3-UTR(s), 5-UTR(s), intron and splice conjugation regions on the β-actin transcript (Femino *et al*. 1998).

smFISH allows us to study interested coding and noncoding genes and RNA at the single-cell level and visualize their subcellular locations robustly and quantitatively. However, it is difficult to synthesize and purify multiple fluorescence labeled oligonucleotide probes, and the hybridization properties might be altered when the target has a high concentration. An improved method was published in 2008. In this procedure, larger amounts of shorter probes (20 nucleotides) that are labeled with single fluorophores at the 3’ end were used (Raj* et al.* 2008) (Fig. 1A). This updated smFISH protocol mitigates the potential off-target effects and false positives from a few probes in the detection of FISH signals. However, only a few nucleic acid molecules can be studied simultaneously owing to the general spectral overlap defect of fluorescent microscopy in single cells.

smFISH combined with branched DNA technology such as RNAscope (Wang* et al.* 2012) and bDNA-smFISH (Battich* et al.* 2013) significantly enhances the intensity of signal, in which genetic target probe, preamplifier, amplifier and fluorescent probes are used to make cascades targeting a particular gene (Fig. 1B). Thousands of human single cells were imaged by bDNA-smFISH with a high ratio of target signal to background noise. For example, smFISH and related methods have been exploited to study the subcellular localizations and cell–cell variability of human lncRNA (Cabili* et al.* 2015), examine the relationship between cellular volume and transcription abundance (Padovan-Merhar* et al.* 2015) and quantitate the 3D mRNA content on tissue of *Drosophila* embryos (Trcek* et al.* 2017).

### Sequential fluorescence *in situ* hybridization (seqFISH)

smFISH has superior resolution but is still limited by target transcripts that can be simultaneously detected. To address this issue, seqFISH (Lubeck* et al.* 2014) improves the multiplexity of target molecules detection by hybridization of multiple rounds of probes. 24 oligonucleotide probes coupled with identical fluorescence are firstly hybridized to each mRNA of fixed cells and imaged *in situ*. Next, the probes are raveled out by DNase I and photobleached to prepare for later rounds. The same steps are repeated using the same group of probes but with different fluorophores in the following hybridization rounds. The sequential color barcode at a fixed locus of the same mRNA finally aligns together. Therefore, after *N* rounds of FISH, each mRNA possesses a unique sequential barcode composition (Fig. 1C). In principle, seqFISH has the capability of distinguishing *N^m^* target molecules using *m* species of probes with *N* rounds of hybridization. seqFISH has been validated by studying 12 mRNA by employing four sets of probes in two rounds (Lubeck* et al.* 2014).

In 2018, the same research group further improved seqFISH to profile the transcription of 10,421 genes. In this study, the author applied seqFISH to intron regions of nascent RNA (Shah* et al.* 2018). 25 primary probes complementary with different sites of target RNA share the same bilateral overhangs, and each overhang consists of two distinct readout sequences which provides the binding places of readout probes. Readout probes labeled with fluorescence are introduced into the reaction and bound with the corresponding overhang sequence on primary probes. In the first four rounds of hybridization, readout probes are removed at each round while primary probes are still hybridized with introns. Three colored readout probes that separately denote three distinct RNA molecules are imaged each time. Therefore 12 targets at different locations with just three colors labeled readout probes are collected after four rounds, which are transformed into 12-pseudocolors due to different combinations of position and color. When five series of four rounds of hybridization are completed, the sequential barcodes of five 12-pseudocolors can identify 12^5^ RNA species in principle (Fig. 1C). It shows the potential to profile the whole transcriptome in single fixed cells.

It is worthy to note that as the hybridization rounds increase, drop out events and imaging crowding occur more frequently which may confound the precise identification of targets.

### Multiplexed error-robustness fluorescence *in situ* hybridization (MERFISH)

Zhuang *et al*. established a highly multiplexed spatial technology called MERFISH with the power to detect and correct errors during hybridization rounds (Chen* et al.* 2015). They skillfully incorporated the superiority of Oligopaints (Beliveau* et al.* 2012) and seqFISH, which markedly increased the efficiency of hybridization and the throughput of gene expression profiles. Another new strategy called binary code of modified Hamming distance (MHD4) was adopted in MERFISH to make this procedure equipped with the robustness of error detection and correction. Two sets of 96 encoding probes are synthesized for a single target gene. Every encoding probe contains a gene target sequence in the center and two readout sequences in the flanks which will bind to diverse complementary readout probes conjugated with a particular fluorophore. Individual gene has a barcode consisting of four types of color. In each round of successive application of *N* readout probes on multiple RNA species, only one kind of readout probe with one color is added and subsequently photobleached. Ultimately, targeted transcripts will produce *N* bites of binary code where “1” marks the successful detection of the signal from hybridized readout probe while “0” means no signal (Fig. 1D). The performance of MERFISH has been confirmed by imaging hundreds and thousands of genes residing in hundreds of single cells (Chen* et al.* 2015). Similar to seqFISH, detection accuracy and efficiency will be influenced as binding rounds go up. Besides, the high cost of probes synthesis and the speed of imaging restrict its wide implementation.

To improve the adaptability of this method, Zhuang *et al*. optimized MERFISH and further expanded the multiplexity in two ways (Moffitt* et al.* 2016). Instead of photobleaching, the readout probes were clipped by a chemical method during successive rounds of smFISH to reduce the scale-dependent imaging time. On the other hand, they extended the microscopic field of view (FOV) to prevent the complex and time-consuming alteration of small FOVs. They implemented this advanced MERFISH on 100,000 cultured cells by imaging the spatial distribution of 140 genes.

In 2018, the same group further merged expansion microscopy into MERFISH, which facilitated the measurement of these molecules with high density compared to MERFISH (Wang* et al.* 2018a). High abundance cDNA is stretched by binding on the expandable gel. The efficiency and accuracy of imaging high concentration molecules boost largely by expansion microscopy. Additionally, this technology is versatile for its compatibility with other ISH and imaging technologies. For instance, branched DNA technology is fused into MERFISH, making it stronger for visualizing shorter RNA species with fewer encoding probes in less exposure time even using lower power laser illumination sources. Remarkably, the size of fluorescent spots and inter-spot brightness variance is not influenced when increasing FISH signal (Xia* et al.* 2019).

### Ouroboros smFISH (osmFISH)

For the purpose of resolving the optical overlap between different fluorescence, one option is to reduce the throughput of genes each time. A similar technology named ouroboros smFISH that discards multiplexed barcoding and amplification identifies a small number of genes in each probe bound round (Codeluppi* et al.* 2018). Therefore, spots of the individual transcripts can be detected accurately without optical distraction from others. After several rounds of continuous hybridization and removal of unique fluorescently probes, a moderate quantity of marker genes’ expression profiles is collected without interference on comparatively low abundance ones. osmFISH can perform on thin larger scale tissue such as the brain section to resolve spatial organization of cell types within the anatomical structure. Although it is designed as a semiautomated pipeline, it is still time-consuming.

### *In situ* sequencing (ISS)

ISS makes directly visualizing and *in situ* sequencing the base pair of target genes a reality. Two strategies are adopted in ISS, including gap-targeted sequencing and barcode-targeted sequencing with padlock probes (Ke* et al.* 2013). In gap-targeted sequencing, padlock probes bind to cDNA that are reversely transcribed from target mRNA with a gap, and cyclized by polymerization and ligation reactions. While in barcode-targeted sequencing, padlock probes perfectly hybridize to cDNA with a barcode sequence on the remaining non-hybridization region, which are circularized only by ligation step. Through rolling circle amplification (RCA) using target primer, rolling-circle products (RCPs) with plenty of replicated target sequences are synthesized finally. Target sequences on the RCP are then sequenced by ligation with the aid of anchor probes which binds to RCP near the target sequences. Subsequently, 9-mer interrogation probe with a fixed 1/4 color representing four natural bases (A/T/C/G) on one random site and eight degenerated bases on the other sites, ligates to anchor probe when its fixed base matches well with the one on the target sequence. Interrogation probes are then imaged and removed for SOLiD (sequencing by oligonucleotide ligation and detection) sequencing of remaining bases on the target sequence (Fig. 1E). Images of the base after several ligations should reflect the real sequence of the target region.

Barcoded anatomy resolved by sequencing (BARseq) combines multiplexed analysis of projections by sequencing (MAPseq) with ISS to achieve the mapping of long-range neuronal projection, which possesses the power to illustrate the spatial anatomy organization of the nerve system by gene expression features *in situ* (Chen* et al.* 2019).

ISS has the ability to read short target sequences. Mutation of nucleotides can be also recognized by gap-targeted sequencing. But RCA has the disadvantages of increasing the size of fluorescent spots which will impede the discrimination of close molecules. It is also inefficient because RNA needs to be reversely transcribed, which may introduce additional technical errors. Recently, some emerging techniques have circumvented this limitation to improve the efficiency of target gene detection, such as BOLORAMIS and SCRINSHOT (Liu *et al*. 2021; Sountoulidis *et al*. 2020), which elegantly utilize barcoded probes that are directly hybridized to RNA and ligated by SplintR ligase to generate RCA copies. However, the number of genes that can be studied in each experiment awaits significant amelioration.

### Fluorescent *in situ* RNA sequencing (FISSEQ)

Researchers have directly sequenced 8102 genes on human fibroblast cells using FISSEQ (Lee* et al.* 2014), which also employs RCA to magnify fluorescent signals of target single molecules. In this method, RNA is reversely transcribed into cDNA using random hexamer primers. cDNA with aminoallyl dUTP cross-links to the matrix through amine reactive linking reaction mediated by PEG. Fixed cDNA is amplified by RCA after circularization to form a single strand nanoball with the size of 200–400 nm in diameter, which similarly cross-links by PEG. Based on sequencing by ligation, partial sequencing is used to sample sequence the RCP in order to minimize the rounds of sequencing but not impair the recognition of each cDNA’s location (Fig. 1F). Nevertheless, FISSEQ needs frequent transition between the small fields of view, and the detection sensitivity is influenced by the increase of detected genes.

### Spatially-resolved transcript amplicon readout mapping (STARmap)

Inherited from ISS, STARmap realizes the 3-dimension (3D) sequencing for intact thick tissue in single-cell resolution. The hydrogel-tissue chemistry methodology is employed in STARmap to maintain the accuracy of multiple detections by stabilizing the location and integrity of amplicons that are produced through enzyme catalyzed RCA (Wang* et al.* 2018b). The pair of probes are introduced to increase the specificity of detected mRNA by lowering the background noise resulting from the mismatches of probes to targets. Padlock probe that can bind to both RNA and modified primers will initiate RCA only when there is correct hybridization of primers with mRNA and padlock probes. Here the approach using padlock probes and modified primers is defined as SNAIL (specific amplification of nucleic acids via intramolecular ligation). The amplicon with numerous repeated 5 nt gene-specific identifier fragments, modified with amine during RCA, is embedded into the hydrogel framework via hydrogel-tissue chemistry. Subsequently, the unlinked proteins and lipids are cleared for enhancing optical transparency and diffusion rate. Notably, the sequencing by ligation is applied to hydrogel-tissue with refined sequencing probes which identifies two bases on the identifier segment in one of five rounds, offering the capacity of sequencing with two-base encoding for error correction (SEDAL). STARmap successfully resolved the distribution and anatomical pattern of different genotypes by sequencing over a thousand mRNA in single-cell on 3D brain tissues.

## TECHNOLOGIES BASED ON ISOLATION THROUGH MICRODISSECTION OR PHOTOREACTION

FISH based technology aims at counting and recognizing partial transcriptomes *in situ* within intact tissues. Alternatively, specifically selecting the regions of interest (ROI) or cells from original tissue also allows us to analyze the spatial profiles at the genome-wide level. Moreover, the advantage of high throughput and sequencing depth enhances the illumination of molecular dynamics of precisely controlled biological processes (Brown* et al*. 2002; van den Brink *et al*. 2020).

### Laser capture microdissection (LCM)

**Figure 2:**
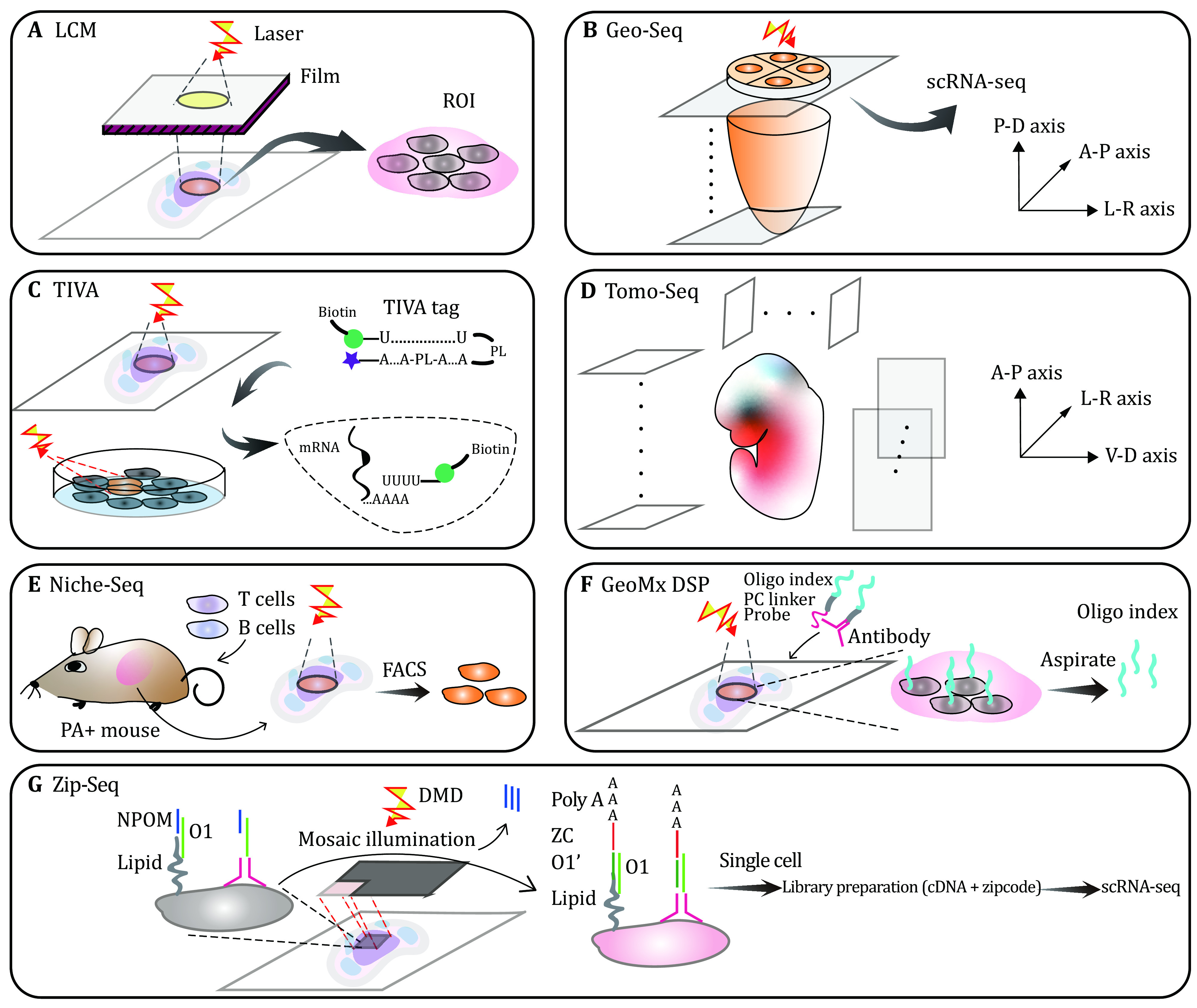
Technologies based on isolation through microdissection or photoreaction. **A** LCM: thermoplastic films covered on frozen tissue sections are activated at interesting regions by laser beam irradiation. The tissue region of interest is isolated along with the film. **B** Geo-seq: embryonic slices along the proximal and distal axis are dissected into ROIs at four quadrants of the anterior-posterior and left-right axis. Each region of interest (ROI) is then analyzed by scRNA-seq. **C** TIVA: TIVA tags with mRNA capture sequence which is sealed by complementary sequence enter cells and are then activated at defined loci of tissue section or culture cells via illumination incises photobleached linkers (PL). Activated tags capture mRNA by released biotin labeled poly(U) sequence. Captured mRNA can be selectively sequenced after biotin affinity purification. **D** Tomo-Seq: identical embryos are sliced into 50–100 sections on the main body axis, respectively. Individual slices are sequenced to reconstruct 3D spatial transcriptome. **E** Niche-Seq: engrafted cells such as T and B cells are injected into photoactivatable reporter transgenic mice to identify histological structures. Immunological cells on the tissue section are photoactivated at specific histological regions. Activated cells are then isolated by FASC. **F** GeoMx DSP: barcoded antibodies and probes containing photocleavable (PC) linker are incubated with targeted mRNA and proteins of cells on the tissue slices. Spatially resolved indexed barcodes are aspirated into microcapillary after PC linkers break via ultraviolet lighting at the target locus of tissues. Oligo barcodes are counted to reflect the abundance of target mRNA and proteins. **G** ZipSeq: cells on the tissue section are labeled with 6-nitropiperonyloxylmethyl (NPOM) caged O1 segment which is introduced through the combination of general cellular membrane antigen with barcoded antibody or lipid insertion into the membrane. Illumination cleaves NPOM, exposing O1 to catch complementary O1’ of zipcoded oligo at selected tissue regions. Target cells are isolated from tissue dissociated single cell suspension and then libraries of zipcode and cDNA from aimed cells are undergone scRNA-seq

LCM is one of the location-directed capturing strategies, which puts the tissues between laser beam and thermoplastic film for accurate isolation of pre-defined cells (Emmert-Buck *et al*. 1996). It begins with mounting frozen tissue on the glass slice followed by regular histological staining. A thin, sterile transparent film with an adhesion layer laid above the tissue slice will be cut by laser beams at predefined locations. Cells in these positions can be separated from the remaining tissues via the focal adhesion force of activated film or adhesive caps, which are then subjected to downstream molecular biological analysis (Fig. 2A). LCM has the capability to interrogate heterogeneous tissues such as tumors and brain, and to scrutinize the transcriptional activity in specific cells in the specific locations of the tissue. LCM also suits for formalin- or alcohol-fixed paraffin-embedded tissues and cellular preparations. But there are several disadvantages of LCM, *e*.*g*., specific cell types are often hardly discerned merely from morphological staining tissue and potential harm to DNA or RNA molecules may happen during laser microdissection.

Immunofluorescent LCM (IF-LCM) can select cells of specific types with the aid of antibody recognition (Murakami* et al.* 2000). To minimize the damage to mRNA when labeling cells with fluorescence in an aqueous solution, IF-LCM labels cells at a rapid speed of just one minute. Labeled cells are microscopely selected and collected by LCM. IF-LCM elucidated the genotype-based gene transcription in a few specific locations of tissues.

Examples of research using LCM grow rapidly. Bandyopadhyay *et al*. constructed the expression profiling of motor neurons in mouse spinal cord by LCM and microarray. This study prompts our understanding of transcriptional changes in spinal cord motor neurons during neurogenic and neurodegenerative diseases (Bandyopadhyay* et al.* 2014). More examples of down-stream utilization of LCM are overviewed elsewhere (Datta* et al.* 2015).

### Geographical position sequencing (Geo-seq)

To bridge LCM with RNA sequencing, Geo-seq and LCM-seq adapted and optimized scRNA-seq on LCM-isolated defined low-input cells (Nichterwitz *et al*. 2016; Chen *et al*. 2017). As a showcase of Geo-seq, Peng *et al*. systematically examined the epiblast cell populations in mid-gastrulation mouse embryos (Fig. 2B). A 3D digital transcriptome data was built up to investigate the continuous transition of gene expression and pathways that models the cell fate segregation and regionalization of germ layer-related stem cells (Peng* et al.* 2016). The merit of Geo-seq is its capability to build a high confidence 3D spatial atlas with serial sections at a resolution of as few as ten cells (Chen* et al.* 2017).

In 2019, Geo-seq was applied to the exploration of the temporal and spatial patterns of code and non-code transcripts in post-implantation mouse embryos (E5.5–E7.5). It successfully revealed the lineage trajectory between germ layers at consecutive time points. This rich data resource of spatiotemporal transcriptomic pattern accelerated our recognition of the regulation of lineage propensity of pluripotent stem cells and identification of core regulatory mechanisms during early embryo development (Peng *et al*. 2019).

However, all these methods based on LCM consume intensive labor and time and the throughput becomes a limiting factor when a large number of samples are surveyed.

### Transcriptome *in vivo* analysis (TIVA)

LCM crudely dissects fixed tissue sections mounted on glass slides so that the natural interaction of cells may be lost. TIVA initiates the non-destructive spatial profiling of gene expression of selected local cells (Lovatt* et al.* 2014). Live cells in culture and tissue section can intake TIVA tag via cell-penetrating peptide (CPP) which will dissociate from TIVA in cytoplasm. Capture oligos labeled with Cy5 and biotin are released from TIVA tag by irradiation. The pair fluorophores can also excite the fluorescence resonance energy transfer (FRET) for confirming the uncaging events. Activated capture oligo catches mRNA in the target cells with photocleavable linkers that are labeled with Cy3. Captured mRNA via biotin affinity purification of lysates are sequenced by RNA-seq (Fig. 2C). As a proof-of-concept, spatially diverse distributions of single neuronal cells were investigated by TIVA.

Although being advantageous as an *in vivo* exploration method, the complexity of TIVA tag and efficiency of conjugation and hybridization still awaits further optimization.

### Tomography sequencing (Tomo-seq)

In addition to laser microdissection, cryosection along interested directions can preserve spatial ordering along latitude or longitude axes. Inspired by this, Tomo-seq in zebrafish embryos acquired 3D developmental regulators and genes with definite spatial patterns in different phases of embryonic morphogenesis (Junker* et al.* 2014). Each of the three embryos was sliced into 50–100 sections according to coordinates of anterior-posterior, left-right and ventral-dorsal body axis respectively, which were eventually reconstructed into an entire 3D embryo image by mathematical algorithms (Fig. 2D). Extracted RNA from different embryonic slices in one axis was reversely transcribed into cDNA using uniquely barcoded poly(T) primer in individual tubes so that all cDNA can be linearly amplified in mixed pools to reduce PCR bias between samples. Finally, a spatial coordinate was approximated through the iterative proportional fitting algorithm. Although the spatial analysis has been improved into 3-dimension with Tomo-seq, the accuracy of genes deduced from inadequate computational fitting is not satisfying. Importantly, the reconstruction requires several morphologically identical samples. All these limitations restrict its application in some rare biological tissues.

### Niche-seq

In order to figure out the rare cell types and their dynamic changes in cellular and molecular levels within the immunological niche, Niche-seq was established in 2017 to apply in live animal or *ex vivo* tissues via the combination of photoactivation (PA) and scRNA-seq technologies (Medaglia* et al.* 2017). A photoactivatable reporter transgenic mouse was intravenously injected with labeled T and B cells to mark the main histological texture of the lymph node. Tissues in different states including normal and infection were photoactivated to express GFP fluorescence within the target regions. FACS were used to enrich specific fluorescent cells followed by single-cell sequencing (Fig. 2E). Various cell types containing scarce populations like NK cells in individual niches were analyzed to illuminate the heterogeneity between immune cell subsets and the dynamic view of the composition of cell types as well as molecular pathways that were induced by treatment. However, the spatial resolution is coarse and the method is limited by available photoactivated colors and transgenic manipulation.

### GeoMx digital spatial profiling (GeoMx Dsp)

Simultaneous profiling of RNA and proteins offers more comprehensive insights than single omics. GeoMx Dsp captures the information of gene expression and protein concentration in particular regions or cell types by utilizing photocleaved oligo barcodes (Merritt* et al.*^ 2019^). RNA probes or protein antibodies coupled with a photocleavable linker that connects to an indexing oligo are incubated with targeted mRNA or proteins on the fixed FFPE tissue slice. At the same time, up to four fluorescence labeled biomarkers can identify the histological structure or cell populations. Regions of interest (ROIs) are selectively illuminated by a digital-micromirror device (DMD) or dual-DMD directed UV light. Photocleaved oligos in targeted areas undergo releasing, gathering by capillary aspiration and dispensing into microplates for final quantitative detection through single-molecule counting nCounter System or RNA-seq (Fig. 2F). Hundreds of RNA and proteins can be simultaneously profiled in enormous ROIs via this powerful method. However, its resolution can hardly reach single cells, and it is not economical when expanding to vast numbers of samples and targets.

### ZipSeq

Instead of obtaining position by isolating specific tissue regions, ZipSeq directly allocates spatial tags (referred to as “zipcode”) on the cell surface at interesting locations through the binding of the high-affinity antibody or the stable lipid insertion into the membrane. The anchor strand contains an overhang sequence (O1) that is caged by thymidine-conjugated 6-nitropiperonyloxylmethyl (NPOM) (Hu *et al*. 2020). Controlled mosaic digital micromirror device (DMD) illumination releases NPOM at selected regions so that the O1 anchor strands will be exposed. Activated local cells are incorporated with special zipcodes by the complementary O1’ strand which hybridizes to the uncaged overhanging sequence of anchor DNA double strands. Besides O1’ sequence, the zipcode strand has an 8 bp barcode for identifying spatial information and a 32 bp poly(A) fragment for amplification in the later step of cDNA library construction. Meanwhile, anchor strands can be labeled with fluorescence in order to filter cells conjugated with the specific antibody. After several rounds of adding distinct zipcodes at specific locations on the tissue section, collected cells are subjected to scRNA-seq analysis to retrieve the zipcode and transcriptome from selected or all cells in target regions (Fig. 2G). The ability of spatial recognization lacks precision due to the area size of defined mosaic pattern.

## ARRAY BASED TECHNOLOGIES

Technologies based on FISH and microdissection usually identify insufficient genes or samples at each run. Even though FISH based spatial transcriptome technologies can directly visualize and even sequence RNA *in situ*, the fluorescent channels can’t satisfy rapid and high-throughput profiling. The down-stream image analysis is complicated, which sometimes requires costly equipment and professional experts. Microdissection related methods have the potential of isolating interested regions and even single cells from tissue slice, however, it is expensive and laborious despite some semiautomated or automated devices that can be integrated. Recently, high-throughput methods based on the spatially barcoded array have been developed to obtain the whole transcriptome of the tissue section and the positional information simultaneously. With the commercialized platform like 10× Genomics Visium, an increasing number of researchers have utilized these convenient platforms to answer many biological and pathological questions. For example, Hou *et al*. probed the transcriptional profiles of the human liver organization via integration of scRNA-seq and Visium (Hou *et al*. 2021). The molecular atlas of specific brain layers was constructed by 10× Genomics Visium to obtain an exquisite reference for position-dependent single cells (Maynard *et al*. 2021). Recently, spatial variances of tumor microenvironment were interrogated during tumor formation, development, metastasis, and a poor curative efficacy was found to correlate with special tumor subclones by 10× Genomics Visium (Nagasawa *et al*. 2021; Zhang *et al*. 2021).

### Spatial transcriptomics (ST)

**Figure 3:**
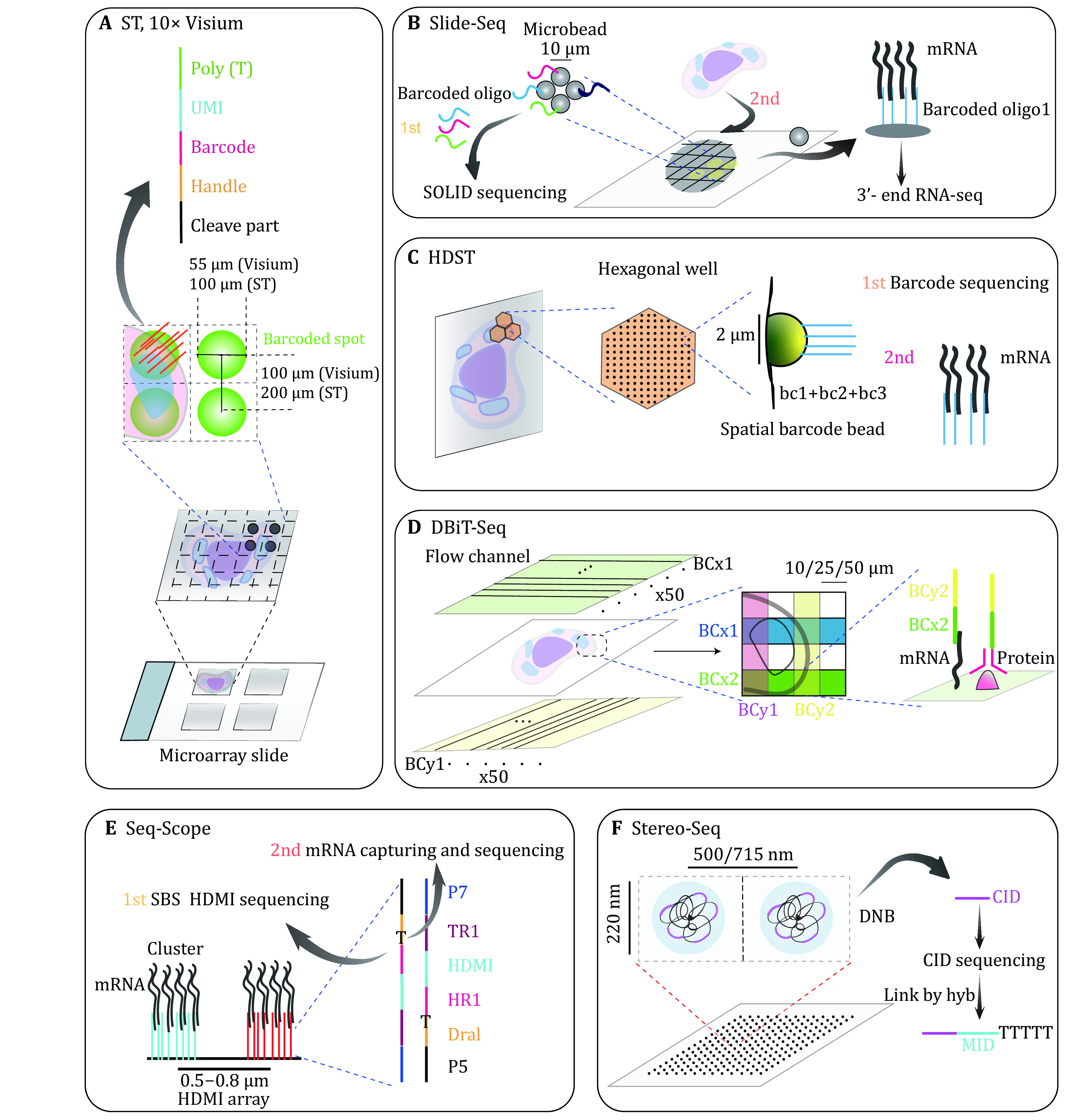
Technologies based on the array. **A** ST and 10× Visium: tissue slice samples are simultaneously sequenced on the same array chip. Thousands of spatially barcoded spots are evenly distributed on the slide. The diameter of the spot is 100 μm (ST) or 55 μm (Visium) and the distance of spot center between two adjacent spots is 200 μm (ST) or 100 μm (Visium) respectively. Each spot has unique spatially barcoded oligo clusters whose poly (T) captures mRNA for later RNA-seq. **B** Slide-Seq: indexed microbeads are randomly organized into monolayer mRNA capture surfaces. The distance between two microbeads is 10–20 μm. Firstly, the spatial index oligo is identified by SOLiD sequencing. Then mRNA can be captured and analyzed spatially through 3’- end sequencing. **C** HDST: 2 μm beads are randomly placed into hexagonal wells on glass slides. Each bead is marked with combinatorial barcodes through split-and-pool with three sets of barcodes. The spatial barcodes are also decoded via seqFISH. mRNA is consequently spatially demultiplexed. **D** DBiT-Seq: two sets of 50 different barcodes flow through microfluidic chips on two perpendicular axes. The tissue section is successively posted on the two chips and labeled with barcode *x* and barcode *y*. The barcode can be coupled with mRNA and proteins when it is conjugated to dT and antibodies. **E** Seq-Scope: molecular clusters are synthesized by the Illumina platform. The distance between the two clusters is 0.5–0.8 μm. Each cluster is composed of plentiful indexed oligos. Single indexed oligo contains P5, Dra1, Oligo-dT, TR1 (TruSeq Read 1), HDMI (high-definition map coordinate identifier), HR1 (HDMI Read 1) and P7. The positions of clusters are confirmed by SBS decoding HDMI and followed by mRNA sequencing. **F** Stereo-Seq: DNA nanoballs (DNB) are stably docked into grid pattern array slide. The diameter of the nanoball is 220 nm and the center-to-center distance is 500 or 715 nm. Each nanoball has multiple identical barcodes which are resolved by sequencing to generate coordinate identifiers (CID). Next, molecular identifiers (MID) and oligo dT are incorporated into CID through hybridization

As a pioneering technology to combine *in situ* RNA amplification and spatially barcoded array, ST prints 1007 pre-defined spots with a size of 100 μm diameter and an inter-spots distance of 200 μm on the 6.2 mm × 6.6 mm array. The spatially barcoded oligo probes consist of cleavage part, sequencing fragment handle, positional barcode, randomized unique molecular identifier (UMI) and mRNA capture poly(T) sequence. Frozen tissue slices are posted on the array and then fixed, HE stained and imaged to obtain the histological images that are subsequently utilized to align the spatially barcoded mRNA in each spot to their corresponding tissue regions. mRNA over the whole tissue section is captured by the poly(T) sequence and polymerizes into RNA-DNA hybrid after mounted tissues are enzymatically permeabilized. The second cDNA strands are synthesized by reverse transcription, followed by library construction and sequencing. Through spatial barcoding and in-array amplification, histological position and gene expression profiling are parallelly registered (Ståhl *et al*. 2016) (Fig. 3A). ST has the strength to unbiasedly delineate gene expression from diverse sample locations for processing simultaneously on the same printed array slides. The manual and automated protocols of ST are both available and the latter is undoubtedly hands-on time saving (Jemt* et al.* 2016; Ståhl *et al*. 2016).

In 2019, 10× Genomics company commercialized ST and further developed it into 10× Genomics Visium technology with increased sensitivity (55 μm diameter spot) and throughput (5000 spots per capture area) (Fig. 3A). Depending on tissue types and sampling thickness, up to 5000 genes from 1–10 cells on average per spatial spot can be obtained. The array-based ST, therefore has broad applications in characterizing different species, organs, developmental stages, biological processes and diseases (Maynard* et al.* 2021; Nagasawa* et al.* 2021). Nevertheless, the cellular resolution has not been readily acquired before more precise printing technology is available.

### Slide-seq

Slide-seq improves the spatial resolution by adapting densely packed bead arrays to capture more subtle tissue features. A monolayer of 10 μm microbeads with specific spatial barcodes are loaded on the rubber-coated glass coverslip. The “puck” surface captures released mRNA from the frozen tissue sections that are mounted onto the bead array. The positional index of each bead can be determined by SOLiD sequencing. The captured mRNA then undergoes library preparation for 3’-end RNA-seq (Fig. 3B). Computational algorithms are also developed for data mining such as non-negative matrix factorization regression (NMFreg) for assigning scRNA-seq identified cell types to beads and finding out spatially non-random genes (Chen* et al.* 2020; Rodriques *et al*. 2019). Most beads are annotated as single cells even though a part of beads have two or more cells. Slide-seq and later updated Slide-seq2 (Chen *et al*. 2020) made enormous progress on the cellular resolution of the spatial transcriptome.

### High-definition spatial transcriptomics (HDST)

HDST makes a step forward to the higher resolution of spatial gene profiling. In HDST, spatially indexed oligos are randomly distributed into the 2 μm beads of hexagonal well on the array. The spatial barcodes are generated by a split-and-pool approach. Similarly, the positional index of barcodes is pre-decoded through sequential fluorophore hybridization and mRNA of the tissue section is sequenced by RNA-seq (Vickovic* et al.* 2019) (Fig. 3C).

With higher spatial resolution obtained, the mRNA capture and detection efficiency on single beads with Slide-seq or HDST remain a bottleneck.

### Deterministic barcoding in tissue for spatial omics sequencing (DBiT-seq)

A simple way to allocate address index to tissue is through deploying individual coordinate parameters on the 2D axis of the tissue section. DBiT-seq labels fixed tissues on the slide with two sets of 50 distinct barcodes which flow through tissue via a microfluidic chip for position recording. Each barcode flowing channel can be set as 10, 25 or 50 µm width (Liu* et al.* 2020) (Fig. 3D), which means that near single-cell resolution can be obtained at the most barcoded areas. However, as the resolution increases, smaller regions of tissue can be recovered with a fixed number of channels. Moreover, the possible risk of lateral spreading of barcodes will cause positioning confusion.

### Seq-scope

Based on the Illumina sequencing platform, Seq-Scope firstly generates HDMI (high-definition map coordinate identifier) clusters of 0.5–0.8 μm spaced apart. Each cluster is composed of plentiful spatially indexed probes. Single indexed oligo contains P5, Dra1, Oligo-dT, TR1 (TruSeq Read 1), HDMI, HR1 (HDMI Read 1) and P7 sequences. The spatial index will be decoded by sequencing by synthesis (SBS). Afterwards, tissue spatial expression profiles at the cellular and subcellular levels can be revealed by the second RNA sequencing step (Cho* et al.* 2021) (Fig. 3E). The efficiency of mRNA capturing has been further improved in comparison with HDST. Therefore, Seq-Scope opens the door of subcellular expression visualization via spatially high throughput sequencing, which holds great promises for implementing high-definition spatial transcriptomic studies in biological and clinical contexts.

Seq-Scope also reputably solved problems that existed in previous array-based methods such as mixed cell types in each dot unit and unclear histological boundary when plotting clustering results onto the spatial position. However, there are some challenges like scratch-related data loss. Besides, more advanced algorithms are needed to extract valuable information at the subcellular level because currently good performance was only obtained with 10 μm cluster grids for reliable cell type classification.

### Spatio-temporal enhanced REsolution omics-sequencing (Stereo-seq)

The BGI company recently developed a method with nanometer resolution and a remarkable capture rate on large frozen tissue sections (Chen* et al.* 2021). Repeated random spatial barcodes DNA nanoball (DNB) of 220 nm of diameter and 500 or 715 nm of inter-distance produced by RCA are docked into the grid-pattern array slide. After imaging and sequencing, the spatial barcode of each DNB, *i*.*e*., the coordinate identifier (CID) can be determined. Molecular identifiers (MID) and oligo-dT are hybridized and ligated to CID containing DNBs which follows mRNA capturing, library preparation and sequencing (Fig. 3F). With its superior capture rate and density, up to 200 mm^2^ tissue sections have been spatially sequenced. As centimeter-scale tissue samples can be analyzed, Stereo-seq greatly facilities spatial transcriptome with a large field of view. Of note, the multiplexing is significantly increased by Stereo-seq so that one can deal with hundreds of samples on continuous slides simultaneously. Technical influences caused by batch operations could also be minimized. However, Stereo-seq can not capture enough mRNA features in each DNB. Besides, it is difficult to distinguish cellular boundary similar to other subcellular spatial approaches. DNB barcode generation and mRNA sequencing on the platform other than BGI sequencer have not been assessed.

Table 1 summarizes the technical metrics of mentioned spatial transcriptomic technologies.

**Table 1:** overview of technical metrics of mentioned spatial transcriptomic technologies

Method	Resolution	Multi- plexity	Gene coverage	Sample	Strength	Limitation
smFISH	Subcellular	Low	Low	Fresh frozen/FFPE	High detection sensitivity	Low throughput
RNAscope	Subcellular	Low	Low	Fresh frozen/FFPE	High resolution and sensitivity	Optical overlap
seqFISH	Subcellular	Low	~100	Fresh frozen	Multiplexity	Predefined probes; drop out error
seqFISH+	Subcellular	Low	10,000	Fresh frozen	Higher multiplexity	Predefined probes; drop out error
MERFISH	Subcellular	Low	Hundreds to thousands	Cultured cells/fresh frozen	Super high resolution; error detection and correction	Predefined probes; detect efficiency and accuracy
osmFISH	Subcellular	Low	Low	Fresh frozen	Low optical overlap	Lower throughput and multiplexity
ISS	Subcellular	Low	Low	Fresh frozen/FFPE	Mutation and isoform detection; reduced background	Optical overlap
FISSEQ	Subcellular	Low	Whole transcriptome	Fresh frozen/FFPE	Genome-wide detection	Impaired sensitivity; time consuming; inefficient
STARmap	Subcellular	Low	Thousands	Thick fresh frozen/FFPE	No RT step; high specificity; error correction	Complexity of image processing; small FOV
LCM-seq/Geo-seq	Single to a few cells	Low	Whole transcriptome	Fresh frozen/FFPE	High depth; tissue compatibility; full-length transcript	Time-consuming; low throughput
TIVA	Single cell	Low	Whole transcriptome	Live cells	*In vivo*; natural state; isolate rare cell types	Complexity of tag; efficiency of conjugation and hybridization
Tomo-seq	Tissue slice	Medium	Whole transcriptome	Fresh frozen	Not requiring specialized equipment	Require similar samples; lack positional details; reconstruction artefact
Niche-seq	Single cell	Low	Whole transcriptome	Live cells/*in vitro* tissue	Isolation of rare immune cells; *in vivo*; natural state	Need transgenic animal
GeoM Dsp	A few cells	Low	Hundreds	Fresh frozen/FFPE	Ability to analyze protein; compatible with fluorescent staining	Throughput; costly
ZipSeq	Single cell	Low	Whole transcriptome	Fresh frozen/FFPE	Ability to analyze protein; combinatorial zipcode	Complexity and hybridization efficiency of combinatorial zipcode; positional details lack
ST/Visium	50/100 μm spot	High	Whole transcriptome	Fresh frozen	High throughput	Not single-cell resolution
Slide-seq	10 μm bead/subcellular	High	Whole transcriptome	Fresh frozen	Improved spatial resolution	Low sensitivity;
HDST	2 μm bead/subcellular	High	Whole transcriptome	Fresh frozen	Subcellular resolution	Low sensitivity
DBiT-seq	10/25/50 μm mosaic grid	High	Whole transcriptome	Fresh frozen/FFPE	Flexibility	Risk of lateral expansion of barcode
Seq-Scope	0.5–0.8 μm distant cluster	High	Whole transcriptome	Fresh frozen	high resolution	Require spatial barcode sequencing
Stereo-seq	220 nm	High	Whole transcriptome	Fresh frozen	Ability for larger tissue section; super high resolution	Require spatial barcode sequencies; detection sensitivity

## SPATIAL MULTI-OMICS

From RNA-seq to scRNA-seq, the heterogeneity of single cells is resolved which gives us the opportunity to gain a profound insight into complicated tissues such as the organization of cell subtypes and dynamic states during biological processes. Comparably, spatial transcriptomics presents a map of molecular architecture in particular tissue regions. Some existing spatial methodologies have shown their strength to grab not only RNA but also proteins and epigenetic information. DBiT-seq profiles whole genome gene expression and proteins of interest at the same time on the frozen fixed tissue (Su* et al.* 2021). Recently, it further demonstrated that epigenome can be analyzed with DBiT-seq pipeline (Deng *et al*. 2021a, b). ExM-MERFISH also could simultaneously detect RNA abundance and the level of protein of interest through hybridization of oligo-conjugated antibodies (Wang* et al.* 2018a). GeoMx Dsp was initially designed to realize the multi-modal information of photocleaved oligo-barcoded RNA and protein content in particular regions or cell types (Merritt* et al.* 2019). The combination of array based spatial transcriptome technology and antibody based protein measurement was developed into spatial multi-omics (SM-Omics) to ensure automatic detection of the transcripts and proteins (Vickovic* et al.* 2020).

Likewise, chromosome organization and interaction with *cis*-elements as well as the dynamic location of active chromatin topology domains can be visualized by the advanced multiplexed FISH platforms such as MERFISH and OligoFISSEQ (Nguyen* et al.* 2020; Su* et al.* 2020) along with gene expression profiles. Now spatial multi-omics approaches have exceeded the incomplete description of cell states merely by transcriptomics and offered multi-modality information for us to explore the accurate identity and characteristics of cell types in various biological contexts. However, the integration of multi-modal measurements requires suitable computational methods. There still needs a lot of work to combine other layers of omics into transcriptomics in the same tissue section, *e*.*g*., metabolomics and epitranscriptome, to enable a holistic understanding of biological processes.

## DATA ANALYSIS

Spatial transcriptomic data should be preprocessed and mined through distinct analytic pipelines dependent on the platform of the sequencing technique. Today there is a mass of available packages and online references for convenient and quick data analysis. A concise summary of spatial analytical methods has been reviewed elsewhere (Longo *et al*. 2021). In most cases, a general single-cell RNA-seq data analysis pipeline can be followed. However, spatial transcriptome data often have different properties, for example, multiple cells could be mixed in one spatial unit leading to potential confounding heterogeneity and segmentation issues, and joint merging of histology images information to enhance spatial gene expression analysis is necessary (Hu *et al*. 2021).

Compared to single cell sequencing, spatially resolved data requires additional spatial barcode demultiplexing to identify the position of transcripts when aligning reads to the genome reference (Navarro *et al*. 2017). Spatial variable genes that are active at specific domains of tissue sections can be computed by a number of open resource packages, such as SpatialDE, SPARK and SPATA (Kueckelhaus* et al.* 2020; Sun* et al.* 2020; Svensson* et al.* 2018). Cell communications within intact tissue texture are explored by ligand–receptor interaction which helps scientists have a better understanding of the interaction of the cells and their microenvironment. Reconstruction of spatial trajectory along undissociated tissue slices can be established at a shared space depending on the gradient changes of expression of distinct cell types (Dries* et al.* 2020; Pham* et al.* 2020; Ren* et al.* 2020; Tran* et al.* 2021), which helps the molecular navigation of space-dependent cell transitions.

To efficiently integrate single-cell and spatial transcriptome data, many existing tools such as bindSC (Dou *et al*. 2020), SPOTlight (Elosua-Bayes *et al*. 2021), RCTD (Cable *et al*. 2021) and Seurat (Stuart *et al*. 2019) are developed through either deconvolution or spatial mapping (Longo *et al*. 2021). Most importantly, analytic algorithms that fully harness histologic images and other tissue architecture modalities will deeply transform the current computational spatial data workflow. Emerging methods such as stLearn and SEDR (Pham *et al*. 2020; Chen *et al*. 2021) that incorporate morphological features and machine learning models have already empowered the exploration of the rich information in spatial transcriptome data.

## SUMMARY AND PERSPECTIVES

Anatomists help us to perceive the composition of our organs and tissues. Histologists clarify the cellular organization and characteristics of tissues. Molecular technologists take a deeper probe into biological substances in cytoplasm and nucleus. While RNA-seq serves as the means to depict a rough picture of mRNA levels of culture cells or tissues on average, the non-negligible cell heterogeneity is then conquered by scRNA-seq. However, cell suspension from intact tissue made it impossible to infer the original anatomical regions and detailed locations. To circumvent this limitation, a variety of spatial transcriptomics technologies have been developed to explore the spatial dimension for comprehensive understanding of the relationship between cellular function and location, the interaction of cell populations and dynamic trajectory of specific subpopulations reacting to stimulus or treatment. These technologies have enabled spatially resolved molecular profiling on almost all types of tissue in model organisms. Studies on pathological samples including diseases and tumors with these spatial omics technologies will undoubtedly uncover the alteration of molecules at important histological sites, leading to the development of novel discoveries for clinical diagnosis, drug design, disease prevention and prognosis evaluation.

Although new methods become available from time to time, no singular spatial transcriptomics technique is currently best for all situations. The limits to resolution and sensitivity, as well as throughput, accessibility, and affordability shall be considered in designing the experimental pipeline. We envision that the integration of multimodal information from single-cell RNA-seq data, spatial multi-omics, and histological features will fully unleash the power of spatial transcriptome to disentangle the complex interplay in both physiological and pathological conditions.

## Conflict of interest

Zhuxia Li and Guangdun Peng declare that they have no conflict of interest.
